# An investigation of multicultural personality traits of EFL learners in English as a medium of instruction setting: The case of Saudi Arabia

**DOI:** 10.3389/fpsyg.2022.1000235

**Published:** 2022-09-16

**Authors:** Talal Musaed Alghizzi, Tahani Munahi Alshahrani

**Affiliations:** Department of English Language and Literature, Imam Mohammad Ibn Saud Islamic University, Riyadh, Saudi Arabia

**Keywords:** cultural empathy, EFL, EMI, emotional stability, multicultural personality, open-mindedness, social initiative

## Abstract

Many studies have investigated the effect of multilingualism on improving personality traits, namely cultural empathy, open-mindedness, flexibility, emotional stability, and social initiative of international students, or students in an international degree program. However, few studies have examined such an issue for EFL learners as they further their academic levels in English as a medium of instruction (EMI) setting. The main tool used in this exploratory descriptive study was the short version (40 items) of the multicultural personality questionnaire (MPQ) that was developed by Van der Zee et al. Five hundred and seventy-seven Saudi female EFL undergraduates participated voluntarily across various academic levels (1–8), as well as recently graduated students; all of whom were exposed to various amounts of EMI. Prior to the study and before administering the questionnaire, the IELTS test was used to measure language proficiency of EFL learners to make sure that the collected data of the MPQ were analyzed statistically to generate means and standard deviations. The results of ANOVA test revealed that not only did all participants improved in cultural empathy followed by open-mindedness, but their improvement of the other dimensions of flexibility, social initiative, and emotional stability appeared to be randomly similar between only two or three groups of academic levels. The results also showed that there was no gradual sequence of developments or deterioration of any multicultural personality dimensions as students advanced in their academic levels or as a result of getting exposed to various amounts of EMI. Finally, this research provides some implications related to widening the scope of the study for further research such as comparing trilingual and bilingual learners and modifying the diagnostic ability of the MPQ.

## Introduction

EFL researchers have acknowledged the bond between the development of multicultural personality (MP) traits—cultural empathy, open-mindedness, flexibility, emotional stability, and social initiative—and learning another language (Cervone and Pervin, 2015). For Dewaele and Botes (2020), these traits are at the top of the hierarchy that comprise a person’s behavior. Reviewing the research on physiological and social factors’ effects on personality showed a positive link between learning a language and improving MP traits (Dewaele and van Oudenhoven, 2009). Based on this stand, we conducted this study within this avenue of research, as multilingualism is a social/cultural variable that is likely to shape a person’s personality (Dewaele and Wei, 2012).

The issue of language, culture, and personality has been the focus of many EFL language researchers (Basow and Gaugler, 2017). Lundell et al. (2018) stated that aspects of MP have been examined in various studies as predictors of university learners’ cultural, behavioral, and academic adjustment. These studies compared international and noninternational students, international employees, and business-major students. Chaika and Zakrenytska (2022) stated that some studies investigated psychological and sociocultural adjustment and the relationship between university adjustment and MP (Van Oudenhoven and Van der Zee, 2002; Van Oudenhoven et al., 2003; Leong, 2007; Anca-Diana et al., 2014; Van Niejenhuis et al., 2018; Bhatti et al., 2019). In addition, other studies (Law et al., 2004; Khatib and Samadi Bahrami, 2013; Dewaele and Al-Saraj, 2015; Dewaele and MacIntyre, 2019; Chaika and Zakrenytska, 2022; Cheraghi and Karamimehr, 2022) have emphasized the relations between MP and emotional intelligence, language proficiency, or anxiety. The mentioned literature provided evidence that no study has characterized EFL learners’ MP development using English as a medium of instruction (EMI) in the Saudi context. Hence, this study involves an exploration of Saudi EFL learners’ MP traits as they further their academic levels. Specifically, we examined how language development affects particular demographic and MP variables. According to our knowledge, no researchers in the EFL field have yet explored the language development and multicultural effectiveness of Saudi EFL learners in universities across academic levels.

## Literature review

### Multicultural personality

Van Der Zee and Van Oudenhoven (2000) offered a definition of multicultural competence (personality) that reflects individuals’ engagement in another culture. According to them, people are culturally competent when they can communicate successfully in another cultural environment, feel comfortable when communicating, and enjoy interacting with people from different cultures. In addition, language theorists have paid considerable attention to the concept of multicultural competence. Whaley and Davis (2007) provided a well-developed definition of multicultural competence, which is an “individual’s ability to function effectively across cultures” (p. 25). Several personality frameworks have been proposed that are related to the cross-cultural domain. In an overview of this concept, Van Der Zee and Van Oudenhoven (2000) were the first to develop a model focusing on multicultural personality. It was mainly developed to evaluate the intercultural effectiveness of international students and expatriates who study abroad and has gradually been extended to different samples, purposes, and contexts. It was established to measure the five cultural personality dimensions: cultural empathy, open-mindedness, flexibility, emotional stability, and social initiative.

Van der Zee and Brinkmann (2004) stated that the most ubiquitous of these dimensions is cultural empathy, which refers to being able to understand the perspectives of people from different cultures and includes items such as “understanding other people’s feelings.” Achieving high scores on this scale means that a person is able to understand the emotions of people from different cultural backgrounds. Another dimension called open-mindedness examines racial and ethnic tolerance between people from different cultures and includes items such as “interest in other cultures.” People who get high scores on this scale appear to be open-minded and have an unbiased attitude toward others’ values, ideas, and traditions. Therefore, Leong (2007) declared that these two dimensions could effectively predict whether a person can behave appropriately in terms of emotion and communication.

Social initiative is another dimension that indicates a person’s ability to establish social relations with people of other cultures. It reflects the person’s behavior in social communications that involve leadership and building social relations. This dimension’s scale includes items such as “ability to speak out” and “leadership.” Therefore, achieving high scores on this scale denotes that a person can easily engage in social relations (Anca-Diana et al., 2014). Emotional stability, another dimension, explains the degree of a person’s calmness in facing cultural problems and differences. It includes factors such as “staying calm under ill luck” and “considering problems solvable” (Hendriks, 2010). The last dimension, flexibility, deals with being able to cope with new situations. It examines how people alter their behavior when facing new and unexpected situations and includes items such as “working according to a plan” and “working according to strict rules.” Thus, achieving high scores on this dimension shows that a person is flexible when dealing with new tasks and situations (Van der Zee and Brinkmann, 2004).

Other researchers have investigated the link between multilingualism and personality traits using the MP questionnaire (MPQ). Dewaele and van Oudenhoven (2009) were the first to investigate how social variables (multilingualism) affect personality traits as measured by the MPQ. The questionnaire also included a question about the number of languages known. Twenty-seven participants were incipient bilinguals (EFL learners), 43 were trilinguals, six were quadrilinguals, and three were pentalinguals. Results revealed that the multilingual group scored significantly higher than the bilinguals on open-mindedness and cultural empathy, but they scored significantly lower on emotional stability. In the same vein, Grin and Faniko (2012) examined the relation between foreign-language skills and intercultural personality, assessing the former using the Common European Frame of Reference for Languages and the latter through the MPQ. The sample participants were 6,434 young Swiss men. Their findings revealed a prevailing relation between three MP dimensions (open-mindedness, cultural empathy, and social initiative) and language skills. Additionally, they found a significant relation between knowing a second language and open-mindedness, social initiative, emotional stability, and flexibility.

### EMI in the Saudi context

Alfehaid (2018) confirmed that implementing EMI partially or completely in teaching EFL learners contributes to the development of English language proficiency to a large extent. EMI can be defined as the use of English as the language of instruction in non-English-speaking countries. The reason to introduce EMI is related to its common advantages among higher-education students. The driving forces for EMI growth in the EFL setting are not associated only with its widely known benefits, but extend to improving graduates’ job prospects and enhancing institutional ranking and global competitiveness (Dimova et al., 2015).

Because of the above-mentioned academic reasons and many other institutional reasons, various researchers have investigated the use of EMI in the Saudi context. For example, Al-Kahtany et al. (2016) conducted research to examine students’ and teachers’ attitudes toward EMI programs. The data collected from questionnaires and structured interviews showed positive attitudes toward the programs concerning their ability to improve EFL students’ four skills of English language learning. In the same vein, Shamim et al. (2016) investigated teachers’ and learners’ experiences and perceptions about the use of EMI in a preparatory year program. The interviews revealed that most teachers and the more proficient students favored the use of EMI.

As for exploring the relation between MP traits and foreign language mastery in the EMI setting, Khatib and Samadi Bahrami (2013) investigated the MP trait development of BA, MA, and PhD EFL learners studying various academic courses using English as the language of instruction. The researchers used the MPQ to measure the learners’ behavioral progress. Results from an ANOVA test revealed that learners’ MP features developed as their academic levels progressed through the BA, MA, and PhD levels. Moreover, Anca-Diana et al. (2014) conducted a study to characterize the MPs of two groups of students to identify their multicultural development. The researchers compared two groups that were studying engineering. One experimental group consisted of EFL learners from various cultures who joined an English-language educational EMI program (*N* = 68). The other was a control group consisting of Romanian students who joined an educational program that used the Romanian language (*N* = 70). Using the MPQ as a tool to measure multicultural traits, the authors noticed that the experimental group scored higher on the MPQ with regard to multicultural adaptation. Additionally, their academic performance was greater than that of the control group.

### Foreign-language mastery and multicultural personality

Al Doghan et al. (2019) believed that as individuals are exposed to a foreign-language context, their multicultural competencies increase. Researchers have recently explored the relationship between foreign-language mastery and MP factors. Matsumoto and Hwang (2013) confirmed that language skills should be linked to multicultural research because multiculturalism requires learners to learn at least one foreign language. According to Onwuegbuzie et al. (2000), demographic and personality variables are essential predictors of foreign-language development. Korzilius et al. (2011) stated that foreign-language mastery could best be assessed by asking participants about the number of foreign languages they speak. Such a self-assessed method would provide sufficient data for analyzing the relation between multicultural effectiveness and language mastery.

Researchers encourage investigating EFL students’ MP traits to examine the relation between multilingualism and the development of MP traits (Dewaele and Botes, 2020). Kağnıcı (2012) stated that since the main goal for university students is to attain high academic performance and pursue qualifications, having a MP is a feature of success that is key to academic performance. Bahrami (2018) asserted that including culture and cultural understanding in the curricula of EFL programs is considered one of the vital requirements for developing MP qualities for EFL learners. According to him, learning a language in isolation from cultural association will not produce successful learning, and learners will not be able to reach the right interpretations of linguistic elements and authentic texts. Bahrami (2012) added that EFL learners face difficulties in communicating with native speakers and establishing meaningful conversations. That is, a second-language speaker may succeed in producing perfect linguistic expressions but fail to understand cultural elements, which may create an inadequate understanding of the foreign language. Thus, Hae-Jung (2010) asserted that EFL learners need to improve their multicultural qualities while learning another language, which will be reflected positively in their behavior. Language researchers have stressed the need for rigorous research that examines MP trait development as well as those traits’ relation to language mastery.

Van Niejenhuis et al. (2018) conducted a recent study to examine the link between learning another language and cultural integration of international students (*N* = 163). They used the MPQ to measure students’ MP traits at two time points (time lag: 3 months). Results revealed that development in L2 proficiency correlated positively with two indicators of cultural integration: identification with the host society and attitudes toward the host culture. Additionally, students with higher levels of open-mindedness achieved higher levels of L2 proficiency development. In another study, Cheraghi and Karamimehr (2022) explored the differences in MP traits between two groups of EFL learners: junior (14–16 years) and senior (16–18 years) high school students. The results of the data collected from the MPQ revealed a significant difference between MP traits of junior and senior high school students in terms of open-mindedness and cultural empathy. To be specific, young EFL learners exhibited higher levels of open-mindedness and cultural empathy. As for other MP traits (social initiative, emotional stability, and flexibility), there was no significant difference between the groups.

### The role of multiculturalism in Saudi vision 2030

Saudi Vision 2030 is a plan developed by the Council of Economic and Development Affairs, led by Deputy Crown Prince Mohammed bin Salman. The purpose of this vision is to develop the economy, create a sovereign wealth fund, reform industry, and encourage tourism. The vision is based on three primary themes: a vibrant society, a thriving economy, and an ambitious nation (Nurunnabi, 2017). This vision is intended to develop the economic, social, and investment domains with long-term goals and to build a flourishing country by offering job opportunities. The development of 80 major projects will inevitably provide more job opportunities for Saudi citizens. Such a vision provides appropriate solutions for unemployment by reinforcing the capabilities of Saudi Arabia’s economic situation. Specifically, Vision 2030’s major goal is to increase private-sector investment to offer more job opportunities for citizens through privatization programs (Media Centre of Saudi Vision, 2019).

In the current study, we analyzed EFL learners’ MPs to predict whether their multicultural traits enable future competence in light of Vision 2030. This particular context necessitates the study of EFL learners’ MP traits. According to the results of related studies, EFL learners may have better chances of employment, with the postulation that multilingualism develops individuals’ positive multicultural traits. Understanding multiculturalism is a crucial step to achieving efficiency for employees. It is extremely important for EFL learners to develop multiculturalism, because they will encounter people of various nationalities and cultural backgrounds in their future careers. Examining EFL learners’ multiculturalism is essential to predicting their multicultural adjustment (Van Der Zee and Van Oudenhoven, 2000). In fact, Hendriks (2010) declared that inadequate proficiency in the language used at work might have a negative impact on employees, which may hinder their productivity. Thus, the MPQ is the most appropriate scale to predict EFL learners’ professional, motivational, and psychological adjustment in a business environment.

The MPQ has been developed as a powerful tool to assess behavior related to motivational, professional, and occupational concerns in a multicultural and international setting. The impact of personality features on developing adaptation in multicultural work has led researchers to investigate the field (Deller, 1997). Competency in both foreign language competency and a MP are considered prerequisites to professionali+sm in the field of internal and international business (Henderson and Louhiala-Salminen, 2011). Kortmann (2016) confirmed that multiculturalism is considered a significant feature of up-to-date business professionalism. The author added, employees have to master certain multicultural skills to succeed in international careers.

Recently, the role of MP in establishing psychological and social adjustment has become the prevailing issue for EFL research. Bahrami (2018) stated that recent research had handled issues related to the MPQ to assess international, national employees, and international students in multicultural settings. These studies have provided evidence for a strong link between psychological adaptation in a multicultural environment and effective MP traits. Korzilius et al. (2011) investigated the relationship between MP dimensions and foreign-language mastery in a business environment. The participants were international and non-international employees of a multinational company. The analysis of the MPQ and demographic information showed that international employees were found to be more open-minded, flexible, and emotionally stable than employees in the non-international group. Kortmann (2016) conducted another recent study to investigate the relationship between multicultural competence, foreign-language mastery, and job performance. The researcher used the MPQ to assess MP traits, questions about the number of spoken languages to measure language mastery, and a self-assessed job performance questionnaire. The participants were nationally and internationally working employees (*N* = 72). Analysis of the results showed that the relation between multicultural competence and language proficiency did not seem to be dynamic. Nevertheless, there was a mutual relation between multicultural competence and job performance in the international employees’ group.

### Research questions

This paper examined the five MP traits of Saudi female EFL learners. Therefore, the study answered the following question:

1. Does multilingualism have an effect on MP traits of EFL female learners in EMI setting at different stages in their education?

Accordingly, the following hypothesis was formulated for the above-mentioned research question:

*H1*: It is hypothesized that EFL students’ MP traits increased as they further their academic learning.

## Materials and Methods

### Research setting and participants

The researchers contacted Students’ Academic Affairs to obtain information about the students who met the following criteria: (a) had completed the preparatory year program requirements, (b) had achieved 50–25 in the Standardized Test For English Proficiency before transferring to the university level, and (c) had never failed, postponed, or withdrawn. The students who conformed to these criteria were contacted to participate in the study voluntarily. Those who agreed to participate were directed to take an adopted free version of the IELTS exam at the college. Then, the IELTS scores were analyzed to measure the students’ language proficiency and place them at the appropriate level. Thus, any progress in MP traits can be attributed to the effect of learning another language with students at the same level of English proficiency. Each level required a specific IELTS score; higher and lower scores were excluded (i.e., Level 1 required a score of 3, and participants who scored more or >3 were excluded). The following Table 1 reveals the participants’ information and IELTS scores required for each level.

**Table 1:** Characteristics of participants.

Academic level	No. of participants	IELTS score
1	31	3
2	56	3.5
3	61	4
4	96	4.5
5	92	5
6	90	5.5
7	68	6
8	49	6.5
9*	34	7
Total	577	

9 = post-university students.

### Instrument

#### Multicultural personality questionnaire

The MPQ is a reliable predictive tool to measure MP traits of foreign language learners (Van Der Zee and Van Oudenhoven, 2000). Researchers have suggested various models to evaluate multicultural competence throughout history. Matsumoto and Hwang (2013) revised ten assessment scales to test multicultural competence and found the MPQ by Van Der Zee and Van Oudenhoven (2000) to be the most suitable test to evaluate how easily people interact and whether they behave properly with people from different cultures. The MPQ was originally developed to establish a measurement tool to evaluate the five cultural personality dimensions: cultural empathy, open-mindedness, flexibility, emotional stability, and social initiative. The original version of the questionnaire comprises 91 items, then reduced to 40, which participants fill out on a Likert scale. Researchers (Van Oudenhoven and Van der Zee, 2002; Van der Zee and Brinkmann, 2004; Leone et al., 2005; Leong, 2007) have examined the short version of the MPQ and found it a valid and reliable tool to measure MP traits. For this study, the questionnaire was transferred into an electronic format (*via* Google Forms). Additionally, to eliminate redundancy, the questionnaire included one extra question on respondents’ academic levels, age, and the number of languages they knew.

### Data collection procedures

The students who agreed to participate in the study, they took the IELTS test to measure their language proficiency and to be placed at the appropriate level. After that, the questionnaire was administered among the students of the department through posters displayed in different places in the college that gave an explanation of the study’s purpose, the time specified for finishing the questionnaire, and a barcode students could scan with their smartphones to answer the questionnaire online. An email was sent to graduates *via* the Student Academic Affairs office. At the beginning of each month (for almost 4 months), the researchers asked their fellow colleagues to remind their students about the questionnaire posters, and a reminder was sent to graduates’ emails. At the end of the semester, the number of respondents reached 577.

### Data analysis

The researchers analyzed the collected data by using SPSS to analyze statistical operations such as means and standard deviations. In addition, The one-way ANOVA test was used to detect differences between academic levels regarding all dimensions. In addition, multiple comparisons (LSD) *post hoc* was employed to investigate which of the specific groups were different. For the students’ MP traits, descriptive statistics were run, and the mean values of their levels of agreement on the five MP traits were interpreted using the criteria shown in Table 2 (Cho and Teo, 2014).

**Table 2:** Criteria for the Interpretation of the mean value of the students’ level of agreement on the MP traits.

Mean value (*x̄*)	Level of agreement	Weighted score
4.21–5.00	Strongly agree	5
3.41–4.20	Agree	4
2.61–3.40	neutral	3
1.81–2.60	disagree	2
0.00–1.80	Strongly disagree	1

### Results

Table 3 shows descriptive statistics for the MP dimensions across all academic levels. Scale means and standard deviations were computed for the MPQ scores for 577 English students at different academic levels. The dimension means were all slightly above the midpoint of the scale (above 3.34). In general, EFL learners’ cultural empathy developed significantly (4.02), followed by open-mindedness (3.77), social initiative (3.61), flexibility (3.59), and emotional stability (3.34).

**Table 3:** Five multicultural personality dimensions of all levels.

	Dimensions					
*N* = 577		*CE*	*OM*	*SI*	*F*	*ES*
	Mean	4.0238	3.7760	3.6146	3.5977	3.3442
	SD	0.56363	0.64464	0.53815	0.76325	0.65299
	Degree	Applicable	Applicable	Applicable	Applicable	Neutral

We used a one-way ANOVA test to detect differences among academic levels regarding cultural empathy. The results in Table 4 revealed a significance value (*p* = 0.029) below 0.05, thus indicating statistically significant differences among academic levels. The table also shows that level one has the highest mean (4.14), followed by level five (4.13). Level three has the lowest mean score (3.85), followed by level two (3.91). Thus, to know which groups differ, we used a multiple comparisons table with the results of the LSD *post hoc* test.

**Table 4:** One-way ANOVA test for cultural empathy with academic levels.

Dimension	Level	Mean	Rank	Std. deviation	*F*	Sig.	Significance
Cultural empathy	1	4.14	1	0.48958	2.156	0.029	Significant at the 0.05 level
	2	3.91	8	0.59243			
	3	3.85	9	0.57772			
	4	4.03	5	0.60451			
	5	4.13	2	0.50237			
	6	3.94	7	0.59435			
	7	4.12	3	0.49483			
	8	4.03	6	0.53203			
	9	4.08	4	0.60202			
Total		4.02		0.56363			

To identify differences among academic levels for cultural empathy, we used the multiple comparisons LSD *post hoc* test. Table 5 included comparisons of one level to each of the remaining levels. When comparing level 1 with level 3, the significance level is 0.017 (*p* = 0.017). This value is less than the 0.05 level required for statistical significance, so these two levels are significantly different. Significant differences also exist for L4 versus L3 (0.043), L5 versus L2 (0.021), L5 versus L3 (0.003), L5 versus L6 (0.026), L7 versus L2 (0.037), and L7 versus L3 (0.006). Other levels show no significant differences in which the significance value is >0.05.

**Table 5:** Statistical results of LSD *post hoc* test for cultural empathy with academic levels.

Academic level (A)^**^	Academic level (B)	Mean difference	Sig.
1	3	0.29475^*^	0.017
4	3	0.18605^*^	0.043
5	2	0.21972^*^	0.021
5	3	0.28002^*^	0.003
5	6	0.18460^*^	0.026
7	2	0.21061^*^	0.037
7	3	0.27091^*^	0.006

* Means the difference is significant at the 0.05 level.

** Column (A) included levels who achieved higher scores compared to the levels in column (B).

Table 6 presents a one-way ANOVA test to examine the differences among academic levels for the open-mindedness dimension. The results in Table 5 reveal no significant differences among groups of different academic levels in this dimension. The significance value (*p* = 0.274) is >0.05, indicating no significant differences among academic levels. The table also shows that level seven has the highest mean score (3.87), followed by level four (3.86). Level three has the lowest mean score (3.60), followed by level one (3.64).

**Table 6:** One-way ANOVA results for open-mindedness with academic levels.

Dimension	Level	Mean	Std. deviation	Rank	*F*	Sig.	Significance
Open-mindedness	1	3.64	0.59549	8	1.238	0.274	Insignificant
	2	3.82	0.63014	3			
	3	3.60	0.63014	9			
	4	3.86	0.66522	2			
	5	3.80	0.67150	4			
	6	3.72	0.61947	7			
	7	3.87	0.53099	1			
	8	3.75	0.71124	6			
	9	3.76	0.78381	5			
Total		3.77	0.64464				

In Table 7, we used the one-way ANOVA test to reveal variances among different academic levels for the social initiative dimension. The significance value (*p* = 0.280) is more than 0.05, revealing no significant differences among academic levels. In addition, the table showed that level four had the highest mean score (3.70), followed by level five (3.67). Level three has the lowest mean score (3.46), followed by level eight (3.55).

**Table 7:** One-way ANOVA results for social initiative with academic levels.

Dimension	Level	Mean	Std. deviation	Rank	*F*	Sig.	Significance
Social initiative	1	3.60	0.59110	5	1.228	0.280	Insignificant
	2	3.64	0.52770	3			
	3	3.46	0.44876	9			
	4	3.70	0.57725	1			
	5	3.67	0.55808	2			
	6	3.57	0.52020	6			
	7	3.62	0.48963	4			
	8	3.55	0.48963	8			
	9	3.58	0.55607	7			
Total		3.61	0.53815				

Table 8 presents a one-way ANOVA test revealing the differences among academic levels for emotional stability. The results demonstrated significant differences among groups of different academic levels because the significance value (*p* = 0.024) is >0.05. The table also shows that level one has the highest mean score (3.59), followed by level two (3.55). Level 9 has the lowest mean score (3.12), followed by level 3 (3.18). Thus, to know which specific groups differ, we used the multiple comparisons table with the results of the LSD *post hoc* test.

**Table 8:** One-way ANOVA results for emotional stability with academic levels.

Dimension	Level	Mean	Std. deviation	Rank	*F*	Sig.	Significance
Emotional stability	1	3.59	0.73931	1	2.224	0.024	Significant
	2	3.55	0.65051	2			
	3	3.18	0.76534	8			
	4	3.44	0.73650	3			
	5	3.38	0.86221	4			
	6	3.27	0.81274	6			
	7	3.21	0.68732	7			
	8	3.34	0.68178	5			
	9	3.12	0.75094	9			
Total		3.34	0.76325				

To identify differences among academic levels for emotional stability, we used a multiple comparisons LSD *post hoc* test. Table 9 compares one level to each of the remaining levels. For level 1 versus level 3, the significance level is 0.015. This value is >0.05 level required for statistical significance, so these two levels are significantly different. Similarly, significant differences exist for L1 versus L6 (0.039), L1 versus L7 (0.020), L1 versus L9 (0.012), L2 versus L3 (0.010), L2 versus L6 (0.030), L2 versus L7 (0.014), L2 versus L9 (0.009), L4 versus L3 (0.043), and L4 versus L9 (0.035). Other levels show no significant differences among them in which the significance value exceeds 0.05.

**Table 9:** Statistical results of LSD *post hoc* test for emotional stability with academic levels.

Academic level (A)^**^	Academic level (B)	Mean difference	Sig.
1	3	0.40825^*^	0.015
1	6	0.32594^*^	0.039
1	7	0.38170^*^	0.020
1	9	0.47545^*^	0.012
2	3	0.36281^*^	0.010
2	6	0.28051^*^	0.030
2	7	0.33627^*^	0.014
2	9	0.43002^*^	0.009
4	3	0.25158^*^	0.043
4	9	0.31878^*^	0.035

* Means the difference is significant at the 0.05 level.

** Column (A) included levels who achieved higher scores compared to the levels in column (B).

In Table 10, we used a one-way ANOVA test to examine differences in flexibility among academic levels. The results demonstrated significant differences among groups of different academic levels because the significance value (*p* = 0.012) is >0.05. The table also shows that level nine has the highest mean score (3.69), followed by level one (3.61). Level three has the lowest mean score (3.34), followed by level six (3.54). Thus, to know which specific groups are different, we used a multiple comparisons table with the results of the LSD *post hoc* test.

**Table 10:** One-way ANOVA results for flexibility with academic levels.

Dimension	Level	Mean	Std. deviation	Rank	*F*	Sig.	Significance
Flexibility	1	3.61	0.58216	2	2.491	0.012	Significant
	2	3.59	0.57960	5			
	3	3.34	0.65388	9			
	4	3.75	0.60342	3			
	5	3.55	0.73922	7			
	6	3.54	0.67119	8			
	7	3.57	0.56028	6			
	8	3.75	0.63328	4			
	9	3.69	0.74442	1			
Total		3.59	0.65299				

Table 11 used a multiple comparisons LSD *post hoc* test to reveal which specific level differs from other levels. It included comparisons of one level to each of the remaining levels. For level 2 versus level 3 comparison, the significance value is 0.036. This value is >0.05 level required for statistical significance, so these two levels are significantly different. Similarly, significant differences exist for L4 versus L3 (0.000), L4 versus L5 (0.032), L4 versus L6 (0.025), L5 versus L3 (0.049), L7 versus L3 (0.040), L8 versus L3 (0.001), and L9 versus L3 (0.011). Other levels revealed no significant differences among them in which the significance value exceeds 0.05.

**Table 11:** Statistical results of LSD post hoc test for flexibility with academic levels.

Academic level (A)^**^	Academic level (B)	Mean difference	Sig.
2	3	0.25154^*^	0.036
4	3	0.41300^*^	0.000
4	5	0.20222^*^	0.032
4	6	0.21354^*^	0.025
5	3	0.21078^*^	0.049
7	3	0.23499^*^	0.040
8	3	0.40779^*^	0.001
9	3	0.35264^*^	0.011

* Means the difference is significant at the 0.05 level.

** Column (A) included levels who achieved higher scores compared to the levels in column (B).

After exploring the descriptives and One-Way ANOVA, (see Tables 3, 4, 6–8, 10), we concluded that the hypothesis with respect to the development of MP traits of EFL learners is rejected. In fact, the results showed that there was no gradual sequence of developments or deterioration of any MP dimensions as students advanced in their academic levels or as a result of getting exposed to various amounts of EMI.

## Discussion

This study attempts to investigate whether multiculturalism has an effect on MP traits of EFL learners using English as the language of teaching academic courses. Based on the research conducted in this field, the MPQ has been recognized as a vital predictive tool to measure MP (Al Doghan et al., 2019). To test our hypothesis, we used data from female EFL learners joining EMI program to track the development of MP traits as they progress in L2 proficiency. The analyses provided no evidence for the role of L2 proficiency in improving EFL learners’ MP traits. As matter of fact, there was no gradual sequence of improvement or decline of any MP traits as learners advanced in their academic levels or as a result of getting exposed to various amounts of EMI.

The results of this study showed some similarities and differences between some of the nine groups. Regardless of students’ academic levels, for example, cultural empathy and then open-mindedness had the highest means for all groups. Such a linear agreement indicates that these EFL learners developed significantly in these two dimensions. Results of other studies in the EMI setting (Khatib and Samadi Bahrami, 2013; Anca-Diana et al., 2014) are in line with this study that show that open-mindedness developed in multilingual learners. Dewaele and van Oudenhoven (2009) commented, “Open-mindedness is a trait that shares characteristics with the lower-order trait Tolerance of Ambiguity that was also found to be higher among people knowing more languages” (p. 10). The justification also could be that students might have been affected by the excessive spread of Saudi Arabia Vision 2030 because it emphasizes these two dimensions. The vision implicitly invites learners to learn to identify and read the behavior, thoughts, and feelings of individuals who belong to different cultural backgrounds. The vision also promotes the capacity to avoid prejudice and be open when encountering people from other cultures with different values and norms.

For the rest of the MP dimensions) flexibility, social initiative, and emotional stability); however, some groups had developed randomly, not according to the sequence of levels. For instance, levels 1, 2, and 4 had higher means in all three other dimensions, organized hierarchically as flexibility, social initiative, and emotional stability. L8 and L9 had high means in only flexibility followed by social initiative, whereas emotional stability remained neutral. Levels 5, 6, and 7 had high means in two dimensions: social initiatives followed by flexibility. For all those levels, emotional stability had not greatly developed. Finally, level 3 developed significantly in the social initiative; however, flexibility and emotional stability improved but not greatly. It becomes very difficult here to justify why such a random sequence of development had occurred. Generally, no continuous advancement or deterioration of the five MP dimensions appeared for all nine academic levels. Instead, the statistically significant differences in the development occurred only between two or five academic levels in only three dimensions: cultural empathy, emotional stability, and flexibility. Thus, although some signs of significant improvement in cultural empathy appeared as participants advanced through their academic levels, some significant decreases also appeared. For example, there were significant increases in the means of L4 compared to L3, in L5 compared to L2 and L3, and in L7 compared to L2 and L3. Significant decreases also appeared for cultural empathy in the means of L3 compared to L1 and in L5 compared to L6. Emotional stability showed a significant increase in the means of L4 compared to L3. However, significant decreases appeared in the means of L3, L6, and L7 compared to L1 and L2 and in the means of L9 compared to L4.

As for flexibility, significant increases appeared in the means of L4, L5, L7, L8, and L9 compared to L3. Significant decreases appeared in the means of L3 compared to L2 and in the means of L5 and L6 compared to L4. These results do against results from previous research (e.g., Grin and Faniko, 2012; Kağnıcı, 2012; Anca-Diana et al., 2014; Dieleman, 2015; Basow and Gaugler, 2017; Van Niejenhuis et al., 2018) that found exposing EFL learners to a foreign language context would greatly improve MP factors. Such a relation between foreign language mastery and MP traits is impressively noticeable in studies related to international students studying abroad or in EFL students joining EMI English programs.

## Conclusion

In short, the results revealed that all EFL participants developed in cultural empathy followed by open-mindedness. However, the improvement of other dimensions of flexibility, social initiative, and emotional stability appeared to be similar for two or three groups of academic levels and different among others. Therefore, no conclusive justifications exist as to why such results occurred. In addition, the results revealed neither gradual developments nor deteriorations in the five MP traits as participants advanced in their academic levels. The significant increases and decreases appeared in only three dimensions and between only two or five academic levels.

Consequently, two possibilities could explain our results. The first possibility is that this study is limited only to bilingual EFL learners in EMI setting from different academic levels and language proficiency levels. Therefore, we recommend that future studies include a more comprehensive sample including bilinguals and trilinguals and apply the MPQ cross-sectionally or longitudinally between one or two genders in similar and different learning environments. The study can be duplicated to compare EFL learners with native language speakers to detect the differences and improvements. Second, the MPQ may be too insensitive to detect changes. In other words, we know the MPQ’s short form aims to increase the validity and reliability of the long version from Van der Zee et al. (2013), but the diagnostic ability of the short-form questionnaire is somehow limited. The MPQ has five Likert-scale designs. The increase or decrease range of the five elements (completely applicable, applicable, neutral, not applicable, and totally not applicable) only include a specific score range (*high*, *low*, or *neutral*). For example, a score range of two groups regarding cultural empathy from 4.21 to 5 would all be described as high cultural empathy, leaving no room for differences. Thus, future studies should increase the MPQ’s sensitivity *via* some modifications. One modification includes another scale of ten for each statement. When participants do the MPQ, they must determine how much a statement applies. These statements include, for example, paying attention to the emotions of others. Participants indicate whether the statement applies, and they must specify *via* numbers how much it does apply.

## Data availability statement

The original contributions presented in the study are included in the article/supplementary material, further inquiries can be directed to the corresponding author.

## Author contributions

All authors listed have made a substantial, direct, and intellectual contribution to the work, and approved it for publication.

## Conflict of interest

The authors declare that the research was conducted in the absence of any commercial or financial relationships that could be construed as a potential conflict of interest.

## Publisher’s note

All claims expressed in this article are solely those of the authors and do not necessarily represent those of their affiliated organizations, or those of the publisher, the editors and the reviewers. Any product that may be evaluated in this article, or claim that may be made by its manufacturer, is not guaranteed or endorsed by the publisher.
